# Crystal structure of AlFe_0.95_

**DOI:** 10.1107/S2414314623010659

**Published:** 2023-12-14

**Authors:** Yibo Liu, Huizi Liu, Changzeng Fan, Wen Bin, Lifeng Zhang

**Affiliations:** a State Key Laboratory of Metastable Materials Science and Technology, Yanshan University, Qinhuangdao 066004, People’s Republic of China; b Hebei Key Lab for Optimizing Metal Product Technology and Performance, Yanshan University, Qinhuangdao 066004, People’s Republic of China; c School of Mechanical and Materials Engineering, North China University of Technology, Beijing, People’s Republic of China; Benemérita Universidad Autónoma de Puebla, México

**Keywords:** crystal structure, B2 phase, Al–Fe system, vacancy

## Abstract

Three B2-type inter­metallic AlFe_1 – δ_ phases (0.18 < δ < 0.05) in the Al–Fe binary system were synthesized by smelting and high temperature sinter­ing methods. The exact crystal structure for δ = 0.05 was refined by single-crystal X-ray diffraction. The amount of vacancy defects at the Fe atom sites was obtained by refining the corresponding site occupancy factor, converging to the chemical formula AlFe_0.95_, with a structure identical to that of ideal AlFe models inferred from powder X-ray or neutron diffraction patterns.

## Abstract 2

Three Fe-deficient B2-type AlFe_1 – δ_ phases have been synthesized, and the exact crystal structure obtained from single-crystal X-ray diffraction measurements is reported for δ = 0.05.

## Graphical abstract

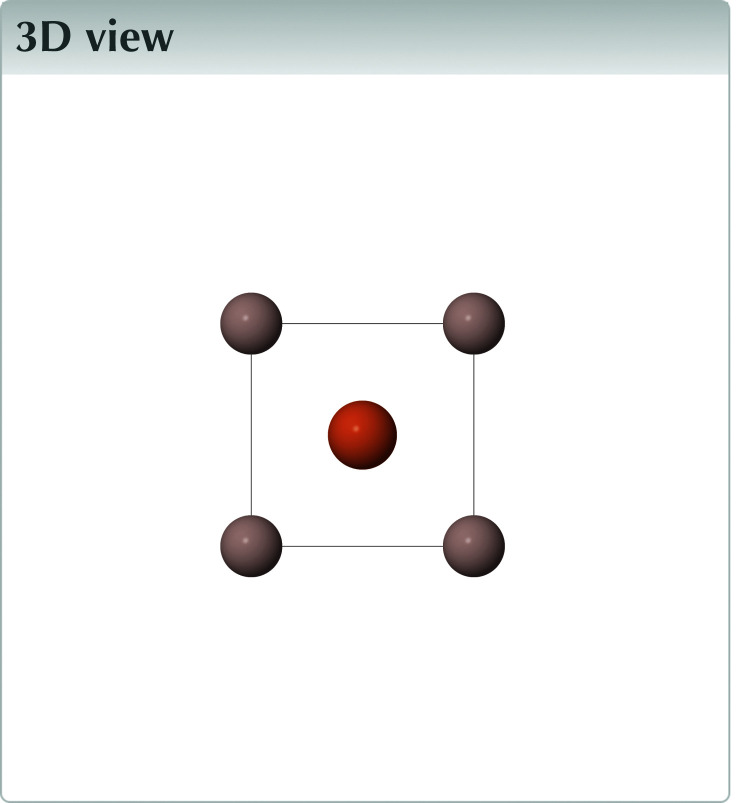

## Structure description

There are diverse inter­metallic phases in the Al–Fe system, among which AlFe has attracted much attention because of its special B2 structure. For example, Van der Kraan & Buschow (1986 ▸) studied the crystal structure of the AlFe phase, after heat treatment at 1273 K for 50 h, by X-ray powder diffraction. The authors suggested that AlFe has a CsCl-type structure with cell parameter *a* = 2.907 Å. During the study of the crystal structure of La(*T*,Al)_13_ (*T* = Fe, Co), a coexisting AlFe cubic phase was discovered. The crystal structure of cubic AlFe was also refined using X-ray powder diffraction data, affording the cell parameter *a* = 2.889 (4) Å, and a model in which Al and Fe atoms are occupying the Wyckoff positions 1*a* and 1*b*, respectively, in space group *Pm* 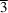 *m* (Guo *et al.*, 1997 ▸). Makhlouf *et al.* (1994 ▸) studied the structure of the magnetic alloys FeAl_1 - *x*_Rh_*x*_ by X-ray diffraction, and concluded that the crystal structure of these alloys remains in the B2 structural type. Stein *et al.* (2010 ▸), using the high-temperature neutron diffraction approach, found that the cell parameter of the AlFe phase gradually increases by increasing the temperature: the cell parameter of the AlFe phase at room temperature, 373, 1353 and 1393 K, is 2.9097, 2.9136, 2.9681 and 2.9720 Å, respectively. They also proposed that the AlFe phase has a B2-type crystal structure (*Pm* 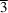 *m* space group, *cP*2 Pearson symbol).

In the present work, three kinds of Fe-deficient B2-type AlFe_1–δ_ phases were synthesized by smelting and high-temperature sinter­ing methods, with very similar lattice parameters. The AlFe_0.95_ (δ = 0.05) phase was obtained by the smelting method, while AlFe_0.82_ and AlFe_0.84_ phases (δ = 0.18 and δ = 0.16) were obtained from an inter­growth sample by the high-temperature sinter­ing method. The refined chemical formula of the AlFe_0.95_ phase is in accordance with the complementary EDX results (see Table S1 of the supporting information). Different options for refinements are listed in Table S2 of the supporting information. The structure description reported herein is for the AlFe_0.95_ (δ = 0.05) phase.

**Figure 1:**
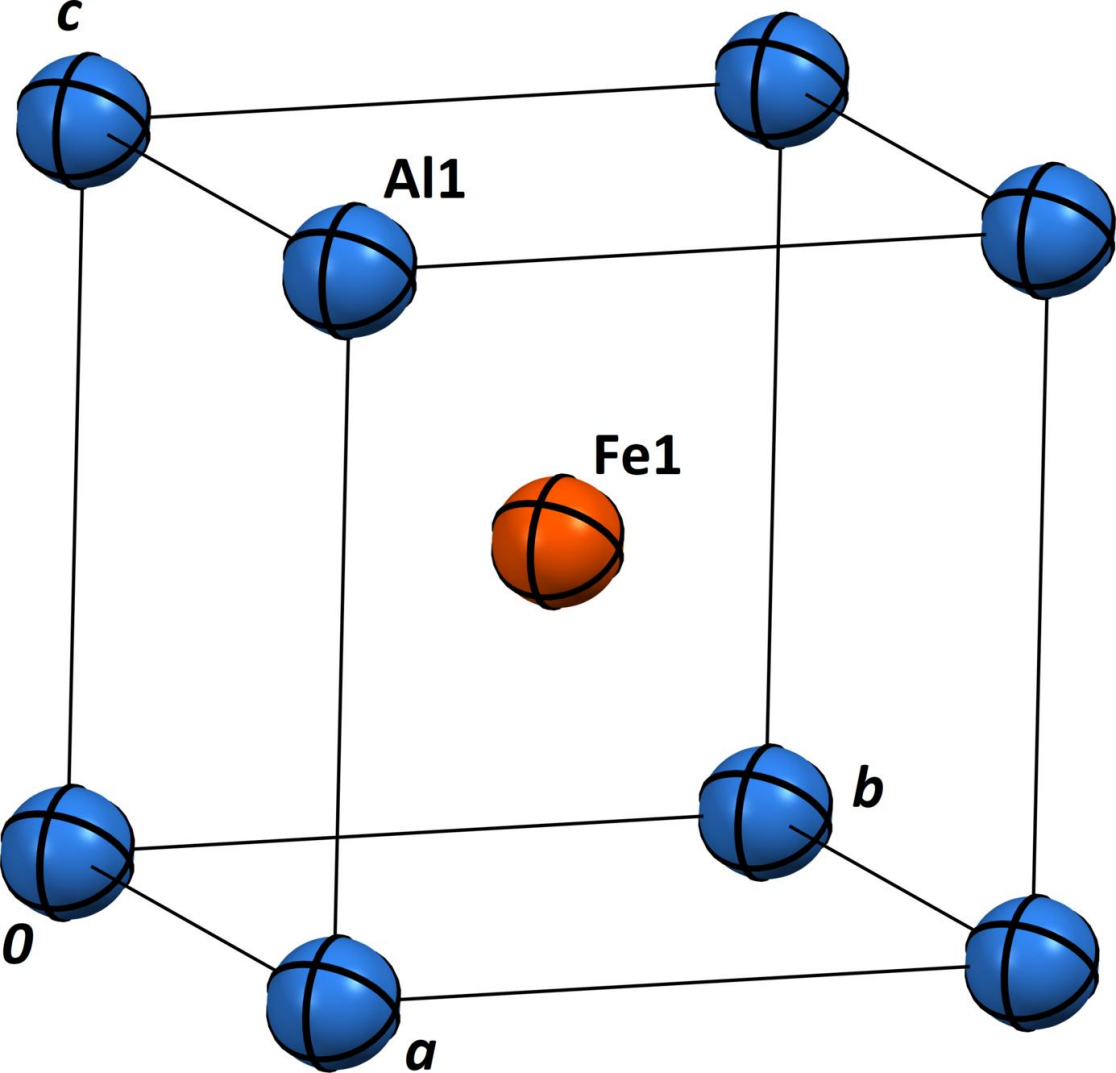
The AlFe_0.95_ structure (one unit cell), with displacement ellipsoids at the 95% probability level.

**Figure 2:**
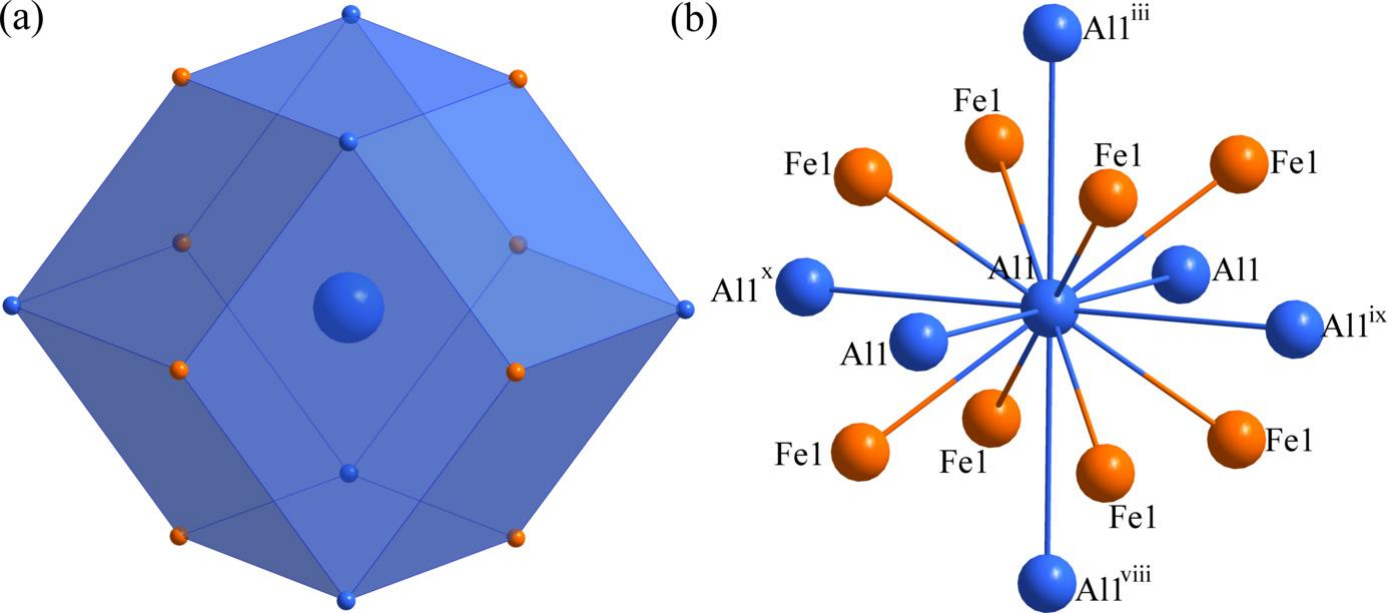
(*a*) The dodeca­hedron formed around the Al1 atom at the 1*a* site and (*b*) the environment of the Al1 atom with displacement ellipsoids given at the 99% probability level. [Symmetry codes: (iii) *x*, *y*, *z* + 1; (viii) *x*, *y*, *z* − 1; (ix) *x* − 1, *y*, *z*; (x) *x*, *y* − 1, *z*.]

**Figure 4:**
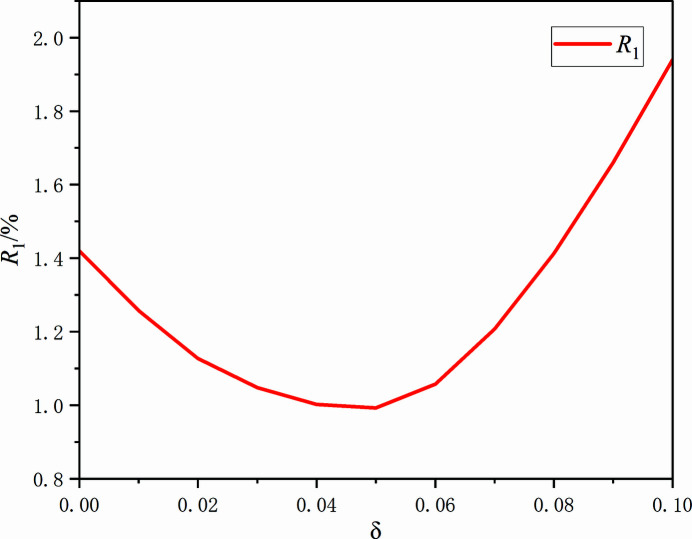
The variation of residual *R*_1_ *versus* δ for the title compound, obtained by refining the model with different values for δ. The minimum of the curve is at δ = 0.05.

Fig. 1 ▸ shows the unit cell of AlFe_0.95_. The environments of the Al and Fe sites are shown in Figs. 2 ▸ and 3 ▸, respectively. The Al1 atom at (0, 0, 0) is centred at a rhombic dodeca­hedron, whose vertices are six Al1 atoms and eight Fe1 atoms; conversely, the Fe1 site at (1/2, 1/2, 1/2) is surrounded by eight Al1 atoms and six Fe1 atoms. The shortest Al1 to Fe1 separation is 2.5164 (4) Å and the shortest Al1 to Al1 link is 2.9057 (5) Å. The *R*_1_ refinement residue *versus* δ values has been plotted for 0 < δ < 0.1 and is shown in Fig. 4 ▸, where one can see that *R*_1_ has the lowest value when the chemical occupancy of Fe atoms is 0.95.

## Synthesis and crystallization

For the here reported sample obtained by smelting (δ = 0.05), high-purity elements Al (indicated purity 99.95%; 1.629 g) and Fe (indicated purity 99.99%; 3.371 g) were mixed in the stoichiometric ratio 1:1 and the alloy was prepared from the elements by arc melting under an argon atmosphere. Suitable pieces of single-crystal grains were broken and selected from the product for single-crystal X-ray diffraction.

For the sample obtained by high-temperature sinter­ing (δ = 0.16 and 0.18), high-purity elements Al (indicated purity 99.95%; 0.7362 g) and Fe (indicated purity 99.9%; 0.2684 g) were mixed in the molar ratio 85:15, ground evenly in an agate mortar, and put into a silicon glass tube, which was vacuum-sealed using a home-made sealing machine. The resulting ampoule was placed in a furnace (SG-XQL1200) and heated up to 473 K for 5 min with a heating rate of 10 K min^−1^, and then heated up to 1373 K for 2 h with the same heating rate. Finally, the sample was slowly cooled to room temperature by turning off the furnace power. Suitable pieces of single-crystal grains were broken and selected from the product for single-crystal X-ray diffraction.

## Refinement

Crystal data, data collection and structure refinement details of AlFe_0.95_ are summarized in Table 1 ▸, while crystal data, data collection and structure refinement details of the AlFe_0.82_ and AlFe_0.84_ phases are summarized in Table S3 of the supporting information. Different options for refinement are listed in Table S2. For the AlFe_0.95_ phase, the maximum and minimum residual electron densities in the final difference map are located 1.30 Å and 0.72 Å from Al1.

## Supplementary Material

Crystal structure: contains datablock(s) I, general. DOI: 10.1107/S2414314623010659/bh4081sup1.cif

Structure factors: contains datablock(s) I. DOI: 10.1107/S2414314623010659/bh4081Isup2.hkl

ESI. DOI: 10.1107/S2414314623010659/bh4081sup3.docx

CCDC reference: 2314099

Additional supporting information:  crystallographic information; 3D view; checkCIF report

## Figures and Tables

**Figure 3:**
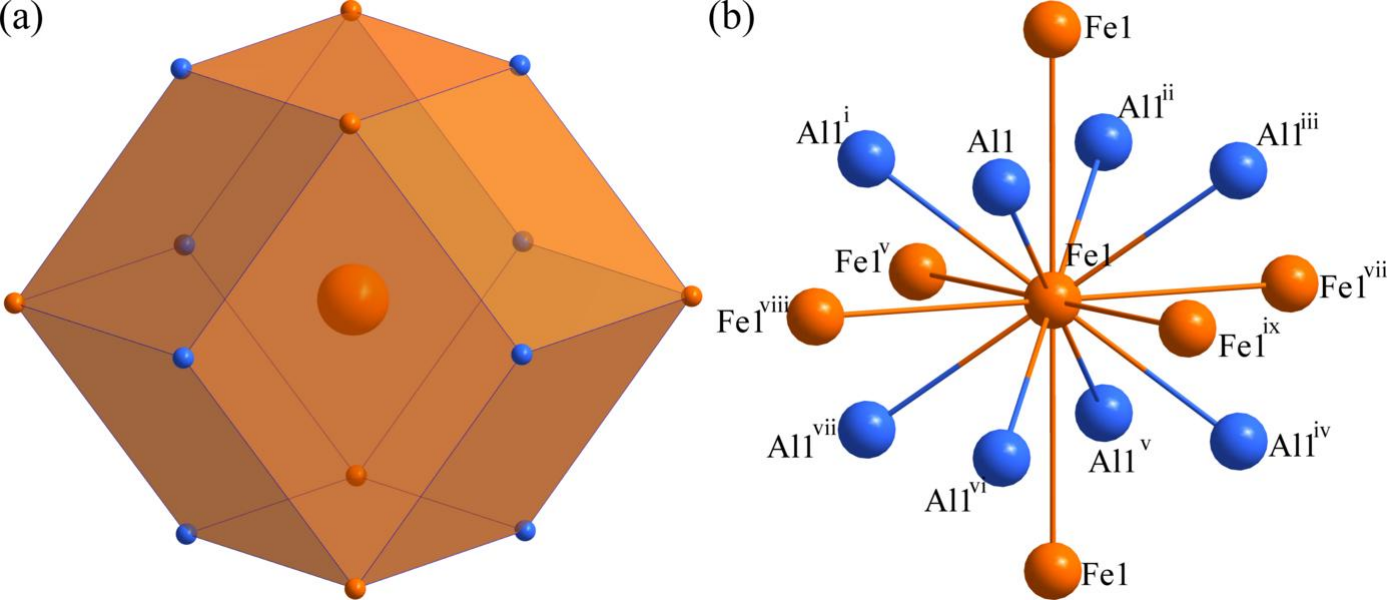
(*a*) The dodeca­hedron formed around the Fe1 atom at the 1*b* site and (*b*) the environment of the Fe1 atom with displacement ellipsoids given at the 99% probability level. [Symmetry codes: (i) *x* + 1, *y* + 1, *z* + 1; (ii) *x* + 1, *y*, *z* + 1; (iii) *x*, *y*, *z* + 1; (iv) *x* + 1, *y* + 1, *z*; (v) *x*, *y* + 1, *z*; (vi) *x*, *y* + 1, *z* + 1; (vii) *x* + 1, *y*, *z*; (viii) *x*, *y*, *z* − 1; (ix) *x* − 1, *y*, *z*.]

**Table 1:** Experimental details

Crystal data	
Chemical formula	AlFe_0.95_
*M*_r_	80.04
Crystal system, space group	Cubic, *P* *m* 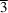 *m*
Temperature (K)	300
*a* (Å)	2.9057 (5)
*V* (Å^3^)	24.53 (1)
*Z*	1
Radiation type	Mo *K*α
μ (mm^−1^)	14.45
Crystal size (mm)	0.10 × 0.08 × 0.06

Data collection	
Diffractometer	Bruker D8 Venture Photon 100 CMOS
Absorption correction	Multi-scan (*SADABS*; Krause *et al.*, 2015 ▸)
*T*_min_, *T*_max_	0.560, 0.746
No. of measured, independent and observed [*I* > 2σ(*I*)] reflections	815, 17, 17
*R*_int_	0.032
(sin θ/λ)_max_ (Å^−1^)	0.709

Refinement	
*R*[*F* ^2^ > 2σ(*F* ^2^)], *wR*(*F* ^2^), *S*	0.010, 0.026, 1.44
No. of reflections	17
No. of parameters	3
Δρ_max_, Δρ_min_ (e Å^−3^)	0.14, −0.24

Computer programs: *APEX3* and *SAINT* (Bruker, 2015 ▸), *SHELXT2014/5* (Sheldrick, 2015*a* ▸), *SHELXL2016/6* (Sheldrick, 2015*b* ▸), *DIAMOND* (Brandenburg & Putz, 2017 ▸), *Mercury* (Macrae *et al.*, 2020 ▸) and *publCIF* (Westrip, 2010 ▸).

## full crystallographic data

### Crystal data

**Table:**

AlFe_0.95_	Mo *K*α radiation, λ = 0.71073 Å
*M**_r_* = 80.04	Cell parameters from 750 reflections
Cubic, *P**m*3*m*	θ = 7.0–30.3°
*a* = 2.9057 (5) Å	µ = 14.45 mm^−^^1^
*V* = 24.53 (1) Å^3^	*T* = 300 K
*Z* = 1	Lump, dark gray
*F*(000) = 38	0.10 × 0.08 × 0.06 mm
*D*_x_ = 5.417 Mg m^−^^3^	

### Data collection

**Table:**

Bruker D8 Venture Photon 100 CMOS diffractometer	17 reflections with *I* > 2σ(*I*)
φ and ω scans	*R*_int_ = 0.032
Absorption correction: multi-scan (SADABS; Krause *et al.*, 2015)	θ_max_ = 30.3°, θ_min_ = 7.0°
*T*_min_ = 0.560, *T*_max_ = 0.746	*h* = −4→4
815 measured reflections	*k* = −4→4
17 independent reflections	*l* = −4→4

### Refinement

**Table:**

Refinement on *F*^2^	0 restraints
Least-squares matrix: full	Primary atom site location: structure-invariant direct methods
*R*[*F*^2^ > 2σ(*F*^2^)] = 0.010	*w* = 1/[σ^2^(*F*_o_^2^) + (0.0141*P*)^2^ + 0.007*P*] where *P* = (*F*_o_^2^ + 2*F*_c_^2^)/3
*wR*(*F*^2^) = 0.026	(Δ/σ)_max_ < 0.001
*S* = 1.44	Δρ_max_ = 0.14 e Å^−^^3^
17 reflections	Δρ_min_ = −0.23 e Å^−^^3^
3 parameters	

### Fractional atomic coordinates and isotropic or equivalent isotropic displacement parameters (Å^2^)

**Table:**

	*x*	*y*	*z*	*U*_iso_*/*U*_eq_	Occ. (<1)
Fe1	0.500000	0.500000	0.500000	0.0084 (3)	0.9499
Al1	0.000000	0.000000	0.000000	0.0086 (4)	

### Atomic displacement parameters (Å^2^)

**Table:**

	*U*^11^	*U*^22^	*U*^33^	*U*^12^	*U*^13^	*U*^23^
Fe1	0.0084 (3)	0.0084 (3)	0.0084 (3)	0.000	0.000	0.000
Al1	0.0086 (4)	0.0086 (4)	0.0086 (4)	0.000	0.000	0.000

### Geometric parameters (Å, º)

**Table:**

Fe1—Al1^i^	2.5164 (4)	Fe1—Fe1^viii^	2.9057 (5)
Fe1—Al1	2.5164 (4)	Fe1—Fe1^v^	2.9057 (5)
Fe1—Al1^ii^	2.5164 (4)	Fe1—Fe1^vii^	2.9057 (5)
Fe1—Al1^iii^	2.5164 (4)	Fe1—Fe1^ix^	2.9057 (5)
Fe1—Al1^iv^	2.5164 (4)	Al1—Al1^iii^	2.9057 (5)
Fe1—Al1^v^	2.5164 (4)	Al1—Al1^viii^	2.9057 (5)
Fe1—Al1^vi^	2.5164 (4)	Al1—Al1^x^	2.9057 (5)
Fe1—Al1^vii^	2.5164 (4)	Al1—Al1^ix^	2.9057 (5)
			
Al1^i^—Fe1—Al1	180.0	Fe1^xi^—Al1—Fe1	180.0
Al1^i^—Fe1—Al1^ii^	70.5	Fe1^xi^—Al1—Fe1^x^	109.5
Al1—Fe1—Al1^ii^	109.5	Fe1—Al1—Fe1^x^	70.5
Al1^i^—Fe1—Al1^iii^	109.5	Fe1^xi^—Al1—Fe1^xii^	70.5
Al1—Fe1—Al1^iii^	70.529 (1)	Fe1—Al1—Fe1^xii^	109.5
Al1^ii^—Fe1—Al1^iii^	70.5	Fe1^x^—Al1—Fe1^xii^	70.5
Al1^i^—Fe1—Al1^iv^	70.529 (1)	Fe1^xi^—Al1—Fe1^xiii^	70.5
Al1—Fe1—Al1^iv^	109.471 (1)	Fe1—Al1—Fe1^xiii^	109.5
Al1^ii^—Fe1—Al1^iv^	109.5	Fe1^x^—Al1—Fe1^xiii^	180.0
Al1^iii^—Fe1—Al1^iv^	180.0	Fe1^xii^—Al1—Fe1^xiii^	109.5
Al1^i^—Fe1—Al1^v^	109.5	Fe1^xi^—Al1—Fe1^viii^	109.5
Al1—Fe1—Al1^v^	70.5	Fe1—Al1—Fe1^viii^	70.5
Al1^ii^—Fe1—Al1^v^	180.0	Fe1^x^—Al1—Fe1^viii^	109.5
Al1^iii^—Fe1—Al1^v^	109.5	Fe1^xii^—Al1—Fe1^viii^	180.0
Al1^iv^—Fe1—Al1^v^	70.5	Fe1^xiii^—Al1—Fe1^viii^	70.5
Al1^i^—Fe1—Al1^vi^	70.529 (1)	Fe1^xi^—Al1—Fe1^ix^	109.5
Al1—Fe1—Al1^vi^	109.471 (1)	Fe1—Al1—Fe1^ix^	70.5
Al1^ii^—Fe1—Al1^vi^	109.5	Fe1^x^—Al1—Fe1^ix^	109.5
Al1^iii^—Fe1—Al1^vi^	70.5	Fe1^xii^—Al1—Fe1^ix^	70.5
Al1^iv^—Fe1—Al1^vi^	109.5	Fe1^xiii^—Al1—Fe1^ix^	70.5
Al1^v^—Fe1—Al1^vi^	70.5	Fe1^viii^—Al1—Fe1^ix^	109.5
Al1^i^—Fe1—Al1^vii^	109.5	Fe1^xi^—Al1—Fe1^xiv^	70.5
Al1—Fe1—Al1^vii^	70.5	Fe1—Al1—Fe1^xiv^	109.5
Al1^ii^—Fe1—Al1^vii^	70.5	Fe1^x^—Al1—Fe1^xiv^	70.5
Al1^iii^—Fe1—Al1^vii^	109.5	Fe1^xii^—Al1—Fe1^xiv^	109.5
Al1^iv^—Fe1—Al1^vii^	70.5	Fe1^xiii^—Al1—Fe1^xiv^	109.5
Al1^v^—Fe1—Al1^vii^	109.5	Fe1^viii^—Al1—Fe1^xiv^	70.5
Al1^vi^—Fe1—Al1^vii^	180.0	Fe1^ix^—Al1—Fe1^xiv^	180.0
Al1^i^—Fe1—Fe1^viii^	125.3	Fe1^xi^—Al1—Al1^iii^	125.3
Al1—Fe1—Fe1^viii^	54.7	Fe1—Al1—Al1^iii^	54.7
Al1^ii^—Fe1—Fe1^viii^	125.3	Fe1^x^—Al1—Al1^iii^	54.7
Al1^iii^—Fe1—Fe1^viii^	125.3	Fe1^xii^—Al1—Al1^iii^	54.7
Al1^iv^—Fe1—Fe1^viii^	54.7	Fe1^xiii^—Al1—Al1^iii^	125.3
Al1^v^—Fe1—Fe1^viii^	54.7	Fe1^viii^—Al1—Al1^iii^	125.3
Al1^vi^—Fe1—Fe1^viii^	125.3	Fe1^ix^—Al1—Al1^iii^	54.7
Al1^vii^—Fe1—Fe1^viii^	54.7	Fe1^xiv^—Al1—Al1^iii^	125.3
Al1^i^—Fe1—Fe1^v^	54.7	Fe1^xi^—Al1—Al1^viii^	54.7
Al1—Fe1—Fe1^v^	125.3	Fe1—Al1—Al1^viii^	125.3
Al1^ii^—Fe1—Fe1^v^	125.3	Fe1^x^—Al1—Al1^viii^	125.3
Al1^iii^—Fe1—Fe1^v^	125.3	Fe1^xii^—Al1—Al1^viii^	125.3
Al1^iv^—Fe1—Fe1^v^	54.7	Fe1^xiii^—Al1—Al1^viii^	54.7
Al1^v^—Fe1—Fe1^v^	54.7	Fe1^viii^—Al1—Al1^viii^	54.7
Al1^vi^—Fe1—Fe1^v^	54.7	Fe1^ix^—Al1—Al1^viii^	125.3
Al1^vii^—Fe1—Fe1^v^	125.3	Fe1^xiv^—Al1—Al1^viii^	54.7
Fe1^viii^—Fe1—Fe1^v^	90.0	Al1^iii^—Al1—Al1^viii^	180.0
Al1^i^—Fe1—Fe1^vii^	54.7	Fe1^xi^—Al1—Al1^x^	54.7
Al1—Fe1—Fe1^vii^	125.3	Fe1—Al1—Al1^x^	125.3
Al1^ii^—Fe1—Fe1^vii^	54.7	Fe1^x^—Al1—Al1^x^	54.7
Al1^iii^—Fe1—Fe1^vii^	125.3	Fe1^xii^—Al1—Al1^x^	54.7
Al1^iv^—Fe1—Fe1^vii^	54.7	Fe1^xiii^—Al1—Al1^x^	125.3
Al1^v^—Fe1—Fe1^vii^	125.3	Fe1^viii^—Al1—Al1^x^	125.3
Al1^vi^—Fe1—Fe1^vii^	125.3	Fe1^ix^—Al1—Al1^x^	125.3
Al1^vii^—Fe1—Fe1^vii^	54.7	Fe1^xiv^—Al1—Al1^x^	54.7
Fe1^viii^—Fe1—Fe1^vii^	90.0	Al1^iii^—Al1—Al1^x^	90.0
Fe1^v^—Fe1—Fe1^vii^	90.0	Al1^viii^—Al1—Al1^x^	90.0
Al1^i^—Fe1—Fe1^ix^	125.3	Fe1^xi^—Al1—Al1^ix^	54.7
Al1—Fe1—Fe1^ix^	54.7	Fe1—Al1—Al1^ix^	125.3
Al1^ii^—Fe1—Fe1^ix^	125.3	Fe1^x^—Al1—Al1^ix^	125.3
Al1^iii^—Fe1—Fe1^ix^	54.7	Fe1^xii^—Al1—Al1^ix^	54.7
Al1^iv^—Fe1—Fe1^ix^	125.3	Fe1^xiii^—Al1—Al1^ix^	54.7
Al1^v^—Fe1—Fe1^ix^	54.7	Fe1^viii^—Al1—Al1^ix^	125.3
Al1^vi^—Fe1—Fe1^ix^	54.7	Fe1^ix^—Al1—Al1^ix^	54.7
Al1^vii^—Fe1—Fe1^ix^	125.3	Fe1^xiv^—Al1—Al1^ix^	125.3
Fe1^viii^—Fe1—Fe1^ix^	90.0	Al1^iii^—Al1—Al1^ix^	90.0
Fe1^v^—Fe1—Fe1^ix^	90.0	Al1^viii^—Al1—Al1^ix^	90.0
Fe1^vii^—Fe1—Fe1^ix^	180.0	Al1^x^—Al1—Al1^ix^	90.0

Symmetry codes: (i) *x*+1, *y*+1, *z*+1; (ii) *x*+1, *y*, *z*+1; (iii) *x*, *y*, *z*+1; (iv) *x*+1, *y*+1, *z*; (v) *x*, *y*+1, *z*; (vi) *x*, *y*+1, *z*+1; (vii) *x*+1, *y*, *z*; (viii) *x*, *y*, *z*−1; (ix) *x*−1, *y*, *z*; (x) *x*, *y*−1, *z*; (xi) *x*−1, *y*−1, *z*−1; (xii) *x*−1, *y*−1, *z*; (xiii) *x*−1, *y*, *z*−1; (xiv) *x*, *y*−1, *z*−1.
