# Supplementary material for: Crystal structure of AlFe0.95
Source: IUCrdata. 2023 Dec 14;8(Pt 12):x231065. doi: 10.1107/S2414314623010659 (PMC10833124; doi:10.1107/S2414314623010659)
Supplement: Supplementary file 3 [file x-08-x231065-sup3.docx]

**SUPPLEMENTARY MATERIALS:**

**Crystal structure of AlFe_0.95_**

**Yibo Liu**^a^**, Huizi Liu**^a^**,** **Changzeng Fan**^ab^***, Bin Wen**^a^ and **Lifeng Zhang**^ac^

^a^ State Key Laboratory of Metastable Materials Science and Technology, Yanshan University,

Qinhuangdao 066004, People’s Republic of China and ^b^ Hebei Key Lab for Optimizing Metal Product Technology and Performance, Yanshan University, Qinhuangdao, Hebei 066004, People’s Republic of China **^c^**School of Mechanical and Materials Engineering, North China, University of Technology, Beijing, People’s Republic of China

*Correspondence email: [chzfan@ysu.edu.cn](mailto:chzfan@ysu.edu.cn)

The chemical compositions were examined quantitatively by energy dispersive X-ray spectroscopy (EDX) analysis attached to a Hitachi S-3400N SEM for the purpose of guiding the crystal structure refinement. The examined points are designated in Fig. S1, and the corresponding results are listed in Table S1. The deviation relative to the results of refinement of chemical composition is probably caused by the tilt of the single crystal surface to the incident beam. In addition, the conductive adhesives and glues may also result in the detected impurity elements of carbon and oxygen. For ease of reading, the atomic ratio of the titled phase was calculated and shown in the last column of Table S1.


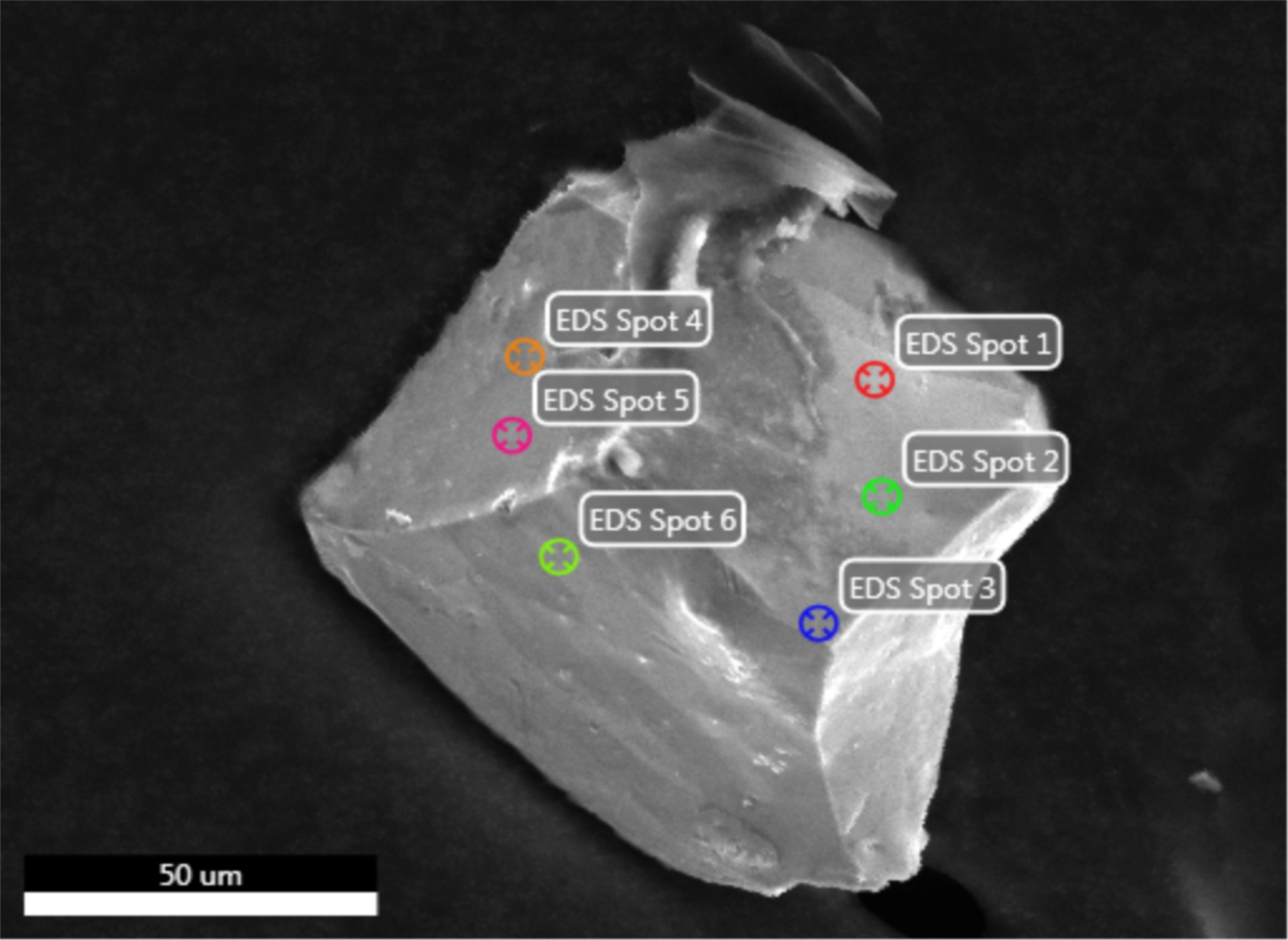


Fig. S1 Single crystal of AlFe_0.95_ with selected spots for EDX analysis

**Table S1 EDX results for selected points as designated in Fig. S1**

|  | Element | Weight (%) | Atomic (%) | Error (%) | Al : Fe |
| --- | --- | --- | --- | --- | --- |
| Spot1 | C K | 18.43 | 43.01 | 11.65 | 1:0.83 |
|  | AlK | 29.92 | 31.08 | 6.51 |  |
|  | FeK | 51.64 | 25.91 | 2.08 |  |
| Spot2 | AlK | 37.30 | 55.18 | 6.95 | 1:0.81 |
|  | FeK | 62.70 | 44.82 | 2.08 |  |
| Spot3 | AlK | 36.03 | 53.82 | 7.01 | 1:0.86 |
|  | FeK | 63.97 | 46.18 | 2.08 |  |
| Spot4 | C K | 17.15 | 41.30 | 11.74 | 1:0.92 |
|  | AlK | 28.48 | 30.54 | 6.67 |  |
|  | FeK | 54.37 | 28.16 | 2.07 |  |
| Spot5 | C K | 21.02 | 47.44 | 11.34 | 1:0.91 |
|  | AlK | 27.41 | 27.54 | 6.55 |  |
|  | FeK | 51.56 | 25.02 | 2.10 |  |
| Spot6 | C K | 31.57 | 65.67 | 9.55 | - |
|  | O K | 3.33 | 5.20 | 11.84 |  |
|  | FeK | 65.10 | 29.12 | 2.17 |  |

**Table S2** Different options of refinement and the resulting refined chemical compositions

|  | location | | compositions | | R1 |
| --- | --- | --- | --- | --- | --- |
| AlFe_0.95_ | 1*a* | 1*b* | Al | Fe |  |
|  | **Al** | **Partial Fe** | **0.99984** | **0.954177** | **0.98** |
|  | Al | Fe | 0.99984 | 0.99984 | 1.59 |
|  | Partial Al | Fe | Occupancy error | |  |
|  | Al | Fe/Al | 1.07144 | 0.928241 | 1.01 |
|  | Al/Fe | Fe | 0.961346 | 1.03833 | 1.08 |
| AlFe_0.82_ | **Al** | **Partial Fe** | **0.99984** | **0.821848** | **1.51** |
|  | Al | Fe | 0.99984 | 0.99984 | 4.80 |
|  | Partial Al | Fe | Occupancy error | |  |
|  | Al | Fe/Al | 1.32256 | 0.677122 | 1.67 |
|  | Al/Fe | Fe | 0.819389 | 1.18029 | 1.47 |
| AlFe_0.84_ | **Al** | **Partial Fe** | **0.99984** | **0.843515** | **3.13** |
|  | Al | Fe | 0.99984 | 0.99984 | 6.03 |
|  | Partial Al | Fe | Occupancy error | |  |
|  | Al | Fe/Al | 1.28948 | 0.710196 | 3.18 |
|  | Al/Fe | Fe | 0.834896 | 1.16478 | 2.83 |

**Table S3** Experimental details of AlFe_0.82_ and AlFe_0.84_ phases

by high-temperature sintering (HTS)

|  | HTS_a | HTS_b |
| --- | --- | --- |
| Crystal data | | |
| Chemical formula | AlFe_0.82_ | AlFe_0.84_ |
| *M*_r_ | 72.88 | 74.09 |
| Crystal system, space group | Cubic, *Pm*$\bar{\text{3}}$*m* | Cubic, *Pm*$\bar{\text{3}}$*m* |
| Temperature (K) | 300 | 300 |
| *a* (Å) | 2.8973 (4) | 2.8920 (5) |
| *V* (Å^3^) | 24.32 (1) | 24.19 (1) |
| *Z* | 1 | 1 |
| Radiation type | Mo-K*α* | Mo-K*α* |
| µ (mm^-1^) | 12.74 | 13.12 |
| Crystal size (mm) | 0.06×0.04×0.04 | 0.06×0.04×0.04 |
| Data collection | | |
| Diffractometer | Bruker D8 Venture Photon 100 COMS | Bruker D8 Venture Photon 100 COMS |
| Absorption correction | multi-scan (SADABS; Krause *et al*., 2015) | multi-scan (SADABS; Krause *et al*., 2015) |
| *T*_min_, *T*_max_ | 0.651, 0.746 | 0.607, 0.746 |
| No. of measured, independent and observed [*I* > 2σ(*I*)] reflections | 912, 14, 14 | 928, 14, 14 |
| *R_int_* | 0.049 | 0.079 |
| (sin θ/λ)_max_ (Å^−1^) | 0.646 | 0.647 |
| Refinement | | |
| *R*[*F*^2^ > 2σ(*F*^2^)], *wR*(*F*^2^), *S* | 0.015, 0.028, 1.46 | 0.031, 0.073, 1.26 |
| No. of reflections | 14 | 14 |
| No. of parameters | 4 | 4 |
| Δ*ρ*_max_, Δ*ρ*_min_ (e Å^−3^) | 0.38, -0.32 | 0.65, -0.57 |

Computer programs: *APEX3* (Bruker, 2015), *APEX3* and *SAINT* (Bruker, 2015), SHELXT 2014/5 (Sheldrick, 2015), *SHELXL2016*/6 (Sheldrick, 2015), *publCIF* (Westrip, 2010).
